# A framework for evaluating long-term impact of newborn screening

**DOI:** 10.1038/s41431-023-01469-8

**Published:** 2023-10-03

**Authors:** Shona Kalkman, Ron A. Wevers, Frits A. Wijburg, Mariska M. G. Leeflang

**Affiliations:** 1 Health Council of the Netherlands, Bezuidenhoutseweg 30, The Hague, The Netherlands; 2 Department of Human Genetics, Translational Metabolic Laboratory (TML), Radboud University Medical Center, Nijmegen, The Netherlands; 3 Department of Metabolic Diseases, Emma Children’s Hospital, Amsterdam University Medical Centers, University of Amsterdam, Amsterdam, The Netherlands; 4 Department of Epidemiology and Data Science, Amsterdam Public Health, Amsterdam University Medical Centers, University of Amsterdam, Amsterdam, The Netherlands

**Keywords:** Population screening, Ethics

## Introduction

Population-based newborn bloodspot screening (NBS) targets treatable conditions that are life-threatening or may cause irreversible damage if treatment is postponed until a diagnosis based on signs and symptoms can be made [1]. The overarching goal of NBS is to achieve substantial health benefit for children with the screened-for conditions [2]. Most countries use a decision-making framework—based on internationally acknowledged criteria—to assess whether the benefits of NBS for a specific condition will sufficiently outweigh the potential harms and burdens of NBS [3, 4]. Recommendations for NBS are based on an informed expectation of the overall balance of benefits and harms, which may not be fully met once the program has been implemented. Benefits and harms of NBS appear to be rarely, if ever, systematically evaluated after implementation. In 2019, the Health Council of the Netherlands recommended to add spinal muscular atrophy (SMA) to the Dutch NBS program, however conditional on a plan for appropriate longer-term evaluation, as there still were considerable uncertainties related to NBS for SMA [5]. In addition, the Council at that time, and for the first time, explicitly recommended that each condition included in the NBS panel should be periodically evaluated [5]. Consequently, the Health Council was asked in 2020 by the State Secretary of Health, Welfare and Sport for advice on what would be needed—criteria, evidence and infrastructure—to enable evaluation of the long-term impact of NBS. Here, we present the subsequent advice, including the reasoning and recommendations made by the Health Council’s Standing Committee on Preconception, Prenatal & Neonatal Screening [6].

### Why newborn screening requires evaluation

The decision to screen for a specific condition is ideally based on unbiased, empirical evidence about the prospective benefits and harms. However, this kind of evidence is lacking for most candidate diseases that undergo initial review for addition to the NBS program. Once a condition meets the criteria for responsible screening, policy-advisers face the question to what extent formal empirical evidence is needed before a decision can be made [7]. While it is inevitable—and to some degree acceptable—to encounter uncertainty during the initial review process, this does not negate the imperative to substantiate anticipated health benefits through scientific evidence. Most often, whether the benefits outweigh the harms and burdens can only be ascertained sometime after the screening has been implemented.

Today, expansion of NBS is accompanied by even more uncertainty about the benefit-harm ratio than before. New pharmacotherapies and technological developments in metabolomics and genomics have led to a substantial expansion of the Dutch NBS panel in a relatively short period of time (see Table 1). These novel tests and treatment options have generally not been assessed in presymptomatic individuals and knowledge on long-term outcome of treatment is lacking. Periodic evaluation would thus create important opportunities for continuous quality improvement; if the benefit-harm ratio is unfavorable then there is a strong case for adjustments or even for removing a condition from the panel [8–10].

**Table 1:** Conditions included in the Dutch newborn bloodspot screening program (as of 1 June 2023).

No.	Condition	Recommended by the Health Council of the Netherlands	Implemented
1	Spinal muscular atrophy (SMA)	2019	2022
2	Mucopolysaccharidosis type I (MPS-I)	2015	2021
3	Severe combined immunodeficiency (SCID)	2015	2021
4	Galactokinase deficiency (GALK)	2015	2020
5	Propionic acidemia (PA)	2015	2019
6	Methylmalonic acidemia (MMA)	2015	2019
7	Carnitine-palmitoyltransferase deficiency type 1 (CPT1)	2015	2019
8	Alpha-thalassemia	2015	2017
9	Beta-thalassemia	2015	2017
–	Carnitine-acylcarnitine translocase deficiency (CACT)	2015	In preparation
–	Carnitine-palmitoyltransferase deficiency type 2 (CPT2)	2015	In preparation
–	Methyl-acetoacetyl-CoA thiolase deficiency (MAT)	2015	In preparation
–	Guanidinoacetate methyltransferase deficiency (GAMT)	2015	In preparation
–	X-linked adrenoleukodystrophy (X-ALD)	2015	Per October 1st, 2023
–	Organic cation transporter 2 defect (OCTN2)	2015	In preparation
10	Cystic fibrosis (CF)	2005	2011
11	Sickle cell disease (SZ)	2005	2007
12	Biotinidase deficiency (BIO)	2005	2007
13	Classic galactosemia (GAL)	2005	2007
14	Medium-chain-acyl-CoA dehydrogenase deficiency (MCADD)	2005	2007
15	Long-chain-hydroxyacyl-CoA dehydrogenase deficiency (LCHADD/MTPD)	2005	2007
16	Very-long-chain-acylCoA dehydrogenase deficiency (VLCADD)	2005	2007
17	3-Methylcrotonyl-CoA carboxylase deficiency (3-MCCD)	2005	2007
18	Glutaric aciduria type 1 (GA-1)	2005	2007
19	HMG-CoA lyase deficiency (HMG)	2005	2007
20	Isovaleric acidemia (IVA)	2005	2007
21	Maple syrup urine disease (MSUD)	2005	2007
22	Multiple carboxylase deficiency (MCD)	2005	2007
23	Tyrosinemia type 1 (TYR-1)	2005	2007
–	Homocystinuria (HCU)	2005	2007; discontinued in 2010 and officially removed in 2016.
24	Congenital adrenal hyperplasia (CAH)	–	2000
25	Congenital hypothyroidism (CH): both thyroidal or central origin	–	1981
26	Phenylketonuria (PKU)	–	1974

### Framework for evaluating impact of newborn screening

In formulating a framework for evaluation of NBS, the Committee initially focused on clarifying the overarching objective to avoid confusion with the decision-making framework regarding inclusion of conditions in the NBS program. According to the Committee, the objective should be to assess that early detection is indeed more beneficial than clinical diagnosis, and that screening is not associated with disproportionate harms and burdens to those screened (benefit-harm ratio). Data from screening programs in other countries may not be generalizable due to differences in screening methods and health care systems. Hence, the benefit-harm ratio can only be ascertained through retrospective analyses of screening-related phenomena observed within the relevant context.

#### Relevant outcomes: benefits, harms and burdens

The Committee defined benefits as substantial and clinically relevant improvements of outcome in patients with the condition detected by NBS when compared to outcomes of non-screened patients who were clinically diagnosed. “Better outcomes” may include decreased mortality, decreased morbidity and increased quality of life in screened populations with the condition. As NBS conditions include metabolic, endocrine and neurological disorders with a broad spectrum of clinical presentations, appropriate outcomes will differ from condition to condition and are best identified by the specialists involved in the care for these particular patient groups, in unison with the patients and parents concerned. For some conditions one core outcome would suffice (for example, intellectual disability for PKU), while for others, the outcomes of interest are manifold and clinically more diverse. Most often, relevant outcomes per condition are already specified as part of the initial assessment of a condition for inclusion in the NBS panel. However, over time other outcomes may become more relevant, or of additional relevance, possibly distinguishing between the short- and longer-term, with a shift from mortality and morbidity to quality of life.

Also, the Committee considered that long-term evaluation should focus on confirming that NBS for a particular condition is indeed not associated with disproportionate harms and burdens to those screened, when weighed against the demonstrated short- and long-term clinical benefits. False-positives, false-negatives and incidental findings are generally undesirable in population screening and would need to be recorded. Close attention should be paid, specifically, to the negative effects of screening for conditions with high degrees of phenotypic variation; this means the same condition can cause a spectrum of clinical symptoms and disease severity in different individuals. Many conditions that are detected by NBS display phenotypic variation. Since the introduction of NBS the numbers of cases have at least doubled compared to the numbers of patients presenting clinically, especially for conditions detected with the sensitive method of tandem mass spectrometry [10]. Phenotypic variation makes it difficult to establish treatment guidelines due to (large) variation in the potential benefit of treatment. In the absence of reliable markers for prognostication, most patients detected by screening will be treated although those with a more attenuated or late onset phenotype may not benefit from (early) treatment. Life-long treatment or dietary regimes that might not have been necessary are a screening-related harm, the extent of which can often not be known at initial review.

#### Collection of evidence

Despite their methodological strengths, the Committee sees little use for randomized study designs. Due to the ultra-low incidence of most NBS conditions, randomized studies will rarely be feasible. Also, once screening for a particular condition has become standard practice, it will be ethically problematic to perform comparative trials, as it would mean withholding screening as a potentially life-saving intervention. Benefits are best captured in observational longitudinal studies in which screened patient cohorts are compared with clinically identified patient cohorts (diagnosed based on symptoms). Clinically identified patient cohorts may consist of historical controls from before the introduction of NBS or patient cohorts in nearby regions without screening but with comparable healthcare systems. The most appropriate follow-up time to assess screening performance will differ between conditions and may vary between a few months or years to a lifetime.

Evaluation of the harms and burdens of NBS requires a different approach, because these effects are more difficult to quantify [11]. The Committee determined that survey research, qualitative studies and expert opinion are probably the best sources to gain insight into the experienced harmful consequences of NBS. An example is a Dutch project on the psychosocial aspects of newborn screening, evaluating parent perspectives on the current program and ongoing expansion [12–14].

### Challenges of periodic evaluation

An important challenge for evaluation of NBS is to ensure a comparable comparison group [10]. Patient groups identified through NBS are not intrinsically comparable with clinically identified patient groups. Screened patients may have better outcomes if screening also identifies people with mild(er) variants of the disease as without screening these people might never have been identified (or much later in life) due to the absence of notable symptoms. The observed benefit may then not necessarily be the result of early detection and treatment but of baseline differences between the two groups. All confirmed cases will be treated but there is heterogeneity of benefit—those with severe disease variants may benefit more than those with milder variants, who might not experience any added benefit at all.

The effect may also be biased if some of the patients already display clinical symptoms at birth and are thus classified as “clinically diagnosed” and excluded from the screened cohort. When screened and non-screened groups are compared, the clinically diagnosed cohort may have poorer outcomes because it (at least in part) may comprise severely affected patients who developed symptoms before NBS took place. Furthermore, when patient groups before and after the introduction of screening are compared, the results may be biased if clinically relevant advancements in diagnostics and treatment over time have not been corrected for [15]. Finally, for some conditions, such as PKU, it will be very difficult to identify a control group as they are already included in screening programs for decades in most developed countries.

Lastly, periodic evaluation would require a national infrastructure for continuous and uniform data collection across different databases and dedicated personnel to record, analyze and publish the data. In the Netherlands, like in most countries, no such infrastructure exists yet. Research efforts in the Netherlands into potential benefits and harms of NBS are often the result of initiatives of individual clinicians or metabolic centers and are only sporadically funded by the Dutch government, which is not very sustainable. The National Institute for Public Health and the Environment (RIVM) does have a database for recording screening results and diagnoses, but it does not contain relevant data on clinical outcomes and there is no central data registry for clinical follow-up. Peer-to-peer consultation also showed that clinician-researchers perceive legal challenges to data sharing and data access (mostly referring to the EU General Data Protection Regulation) obstructing research on long-term benefits and harms of NBS.

### Recommendations

The Committee stressed the importance of a sustainable program for short and long-term follow-up of patients identified through NBS, by collecting data on (clinically) relevant, core outcome measures. Such a program should encompass data concerning both the beneficial as well as harmful effects of NBS on the screened individuals.

To inform policy-making about the long-term impact of NBS, the Committee advised to invest in data collection and data availability for research. Following screened and non-screened groups for a longer time could provide valuable insight into long-term benefits not only in terms of morbidity and mortality, but also quality of life. Since the feasibility of randomized trials is severely limited, observational cohort studies are the most reasonable alternative. To capture harms and burdens, follow-up and data collection would need to extend beyond patients confirmed to have the condition. Long-term data collection and research into a large variety of outcomes poses methodological and operational challenges and will entail considerable time and economic investments. Without truly comparable comparison groups, policymakers will need to be aware of potential biases when interpreting the data.

Given the constraints on public resources, the Committee recommended prioritizing research for those conditions on which doubt has been raised about the benefit-harm ratio (e.g., 3-methylcrotonyl-CoA carboxylase deficiency) [16] and, more in general, conditions with a paucity of data on longer-term clinical outcome. Prespecifying the relevant core outcomes and timelines for decision-making per condition will help to narrow down the scope of research efforts [17]. The input of clinicians, parents and other patient advocates is essential to restrict data collection and timelines to the outcomes that are truly meaningful. To reliably inform local screening policies, data collection would need to be uniform and screening practices as comparable as possible. International collaboration, for example within Europe, would enable more or quicker evidence generation, especially for rarer conditions and for countries with similar health care systems. Recognizing that assessing the long-term impact of NBS demands both political commitment and financial resources, the Committee ultimately determined that decision-makers must prioritize acquiring more comprehensive data about its effectiveness. This approach will significantly enhance the rationale and sustainability of the entire program.
