# Correction: A framework for evaluating long-term impact of newborn screening

**DOI:** 10.1038/s41431-023-01501-x

**Published:** 2023-11-29

**Authors:** Shona Kalkman, Ron A. Wevers, Frits A. Wijburg, Mariska M. G. Leeflang

**Affiliations:** 1 Health Council of the Netherlands, Bezuidenhoutseweg 30, The Hague, The Netherlands; 2 Department of Human Genetics, Translational Metabolic Laboratory (TML), Radboud University Medical Center, Nijmegen, The Netherlands; 3 Department of Metabolic Diseases, Emma Children’s Hospital, Amsterdam University Medical Centers, University of Amsterdam, Amsterdam, The Netherlands; 4 Department of Epidemiology and Data Science, Amsterdam Public Health, Amsterdam University Medical Centers, University of Amsterdam, Amsterdam, The Netherlands

**Keywords:** Population screening, Ethics

Correction to: *European Journal of Human Genetics* 10.1038/s41431-023-01469-8, published online 03 October 2023

The article “A framework for evaluating long-term impact of newborn screening”, written by Kalkman et al., was originally published electronically on the publisher’s internet portal on 3 October 2023 without open access. With the author’ decision to opt for Open Choice the copyright of the article changed on 11 November 2023 to © Author(s) 2023 and the article is forthwith distributed under a Creative Commons Attribution 4.0 International License, which permits use, sharing, adaptation, distribution and reproduction in any medium or format, as long as you give appropriate credit to the original author(s) and the source, provide a link to the Creative Commons licence, and indicate if changes were made. The images or other third party material in this article are included in the article’s Creative Commons licence, unless indicated otherwise in a credit line to the material. If material is not included in the article’s Creative Commons licence and your intended use is not permitted by statutory regulation or exceeds the permitted use, you will need to obtain permission directly from the copyright holder. To view a copy of this licence, visit http://creativecommons.org/licenses/by/4.0.

The original article has been corrected.

